# The Challenge of Misleading Information: Does the Interaction between Zinc and Vitamin D Influence the Immune Response against SARS-CoV-2 in the Elderly Population?

**DOI:** 10.3390/life14101277

**Published:** 2024-10-08

**Authors:** Deise Maria Rego Rodrigues Silva, Pedro Henrique Macedo Moura, Rajiv Gandhi Gopalsamy, Eloia Emanuelly Dias Silva, Marina dos Santos Barreto, Ronaldy Santana Santos, Pamela Chaves de Jesus, Jessiane Bispo de Souza, Lucas Alves da Mota Santana, Adriana Gibara Guimarães, Lysandro Pinto Borges

**Affiliations:** 1 Department of Pharmacy, Federal University of Sergipe, São Cristóvão 49100-000, SE, Brazil; phmm694@gmail.com (P.H.M.M.); sbarretomarina@outlook.com (M.d.S.B.); ronaldyss19@gmail.com (R.S.S.); pamcjesus@outlook.com (P.C.d.J.); jeisse.nik@hotmail.com (J.B.d.S.); adrianagibara@hotmail.com (A.G.G.); 2 Department of Biosciences, Rajagiri College of Social Sciences, Kalamaserry, Kochi 683 104, Kerala, India; egarajiv@gmail.com; 3 Department of Biology, Federal University of Sergipe, São Cristóvão 49100-000, SE, Brazil; eloiaemanuelly@gmail.com; 4 Graduate Program in Dentistry, Federal University of Sergipe, São Cristóvão 49100-000, SE, Brazil; lucassantana.pat@gmail.com; 5 Department of Immunology, Institute of Biomedical Sciences, University of São Paulo, São Paulo 05508-000, SP, Brazil

**Keywords:** antibodies, COVID-19, immunization, neutralizing, vitamin D, SARS-CoV-2, zinc

## Abstract

Immunization is a challenge for the elderly population and can leave this group more vulnerable to opportunistic pathogens such as SARS-CoV-2. Due to this situation, while vaccines were in the development phase, hypotheses were raised about the role of vitamins and minerals in immunization. In Brazil, there was a controversy regarding the well-known COVID-19 Kit, a standardized prescription for positive cases that contained zinc, and vitamin D, and anti-parasitic drugs. There was great controversy in scientific circles, since COVID-19 brought a major challenge for health professionals and public authorities: misleading information. In this study, we evaluated the role of vitamin D and zinc in the production of anti-SARS-CoV-2 neutralizing antibodies (NAbs) in a group of elderly residents in a nursing home in northeastern Brazil. Serum levels of COVID-19 NAbs were assessed, along with vitamin D and zinc, in two phases. The first (T1) was in August 2022 with 26 elderly people, and the second (T2) was in March 2023 with 21, due to the death of five participants. Overall, we observed satisfactory levels for vitamin D, with no participants showing a deficiency in either test, and zinc, with only two participants having a negative result at T1 and three at T2. However, a drop in the average number of NAbs was observed, especially in women (T1 = 89 ± 19 vs. T2 = 57 ± 44), highlighting the importance of monitoring this immunological parameter in the population studied. Based on the results, we suggest that there is no synergism between the micronutrients studied and NAbs (*p* > 0.05). Further studies are needed to consolidate the findings of an absence of synergism between vitamin D and zinc in the maintenance of NAbs.

## 1. Introduction

The COVID-19 pandemic, declared by the World Health Organization (WHO) in 2020, has had a significant impact on the elderly population. Worldwide, the pandemic has resulted in more than 750 million confirmed cases and almost 7 million deaths [1]. In Brazil, COVID-19 has had a major impact, with nearly 7 million cases and more than 700,000 deaths [2]. More than 80% of deaths worldwide were associated with the elderly (over 60 years of age) [3], and in Brazil, it is estimated that the elderly population accounted for 76.6% of deaths [1]. To minimize the impact on this population, the elderly group was classified as a priority group in the vaccination queue in Brazil and was able to receive vaccine doses in January 2021 [4].

The elderly has been considered a risk group and are more likely to develop serious complications and death than the younger population [4]. In the search for ways to protect this population against SARS-CoV-2 while vaccines have been developed, research has been initiated on the influence of vitamins and minerals on the coronavirus through the effect they can have on the immune system through supplementation [5]. Due to the lack of information about effective treatments for COVID-19, and in search of a way to minimize its impact on public health, the COVID-19 Kit was created in Brazil. This kit consisted of anti-parasitic drugs, antibiotics, anti-inflammatories, vitamins such as vitamin C and D, and minerals such as zinc. There was a great deal of scientific and political discussion about these prescriptions, since there were no studies to demonstrate the effectiveness of this kit against COVID-19 [6,7,8].

Vitamin D (Vit D) is a fat-soluble vitamin and/or hormone that plays a key role in bone health and calcium homeostasis. This vitamin is effective in stimulating the innate and adaptive immune systems [9,10]. This may be due to Vit D’s positive regulation of the antimicrobial peptide cathelicidin (LL-37). This peptide is involved in inhibiting bacteria by disrupting the cell membrane and affects certain viruses and fungi. LL-37 is also responsible for promoting chemotactic, immunomodulatory effects and affects serum levels of pro-inflammatory cytokines, as well as increasing autophagy in macrophages and promoting innate immunity against viral infections [11]. In addition, Vit D plays a role in suppressing the production of pro-inflammatory cytokines, including modulating the production of T and B lymphocytes [9,12]. Lymphocytes are also directly involved in the fight against infection by recognizing antigens and activating macrophages, as well as in immunological memory by producing antibodies that neutralize viral access to host cells [13]. These antibodies are known as neutralizing antibodies (NAbs), which are also produced by B lymphocytes and are associated with the primary blocking of various viral proteins involved in the viral infection mechanism [13].

In addition to vitamins, the metal ion zinc (Zn) is another important element in tissue homeostasis and in immune and inflammatory processes [14]. Trace elements play an important role in maintaining good health because of their influence on the stimulation of immunity, and they can interfere with the modulation of immune cells [14]. Therefore, supplementation with this ion is associated with the maintenance of immunity, leading to an increase in the synthesis of pro-inflammatory cytokines such as tumor necrosis factor (TNF-α), interleukin-1β (IL-1β), and interleukin (IL)-6 (IL-6) [15]. For adaptive immunity, Zn deficiency can lead to a reduction in CD8+ T-cell responses and the activation of T-helper cells with thymic atrophy. This leads to a reduction in the secretion of thymulin, a thymic hormone produced by epithelial cells that is involved in the maturation and differentiation of T cells [16].

NAbs against SARS-CoV-2 can be stimulated by viral exposure or vaccination, regardless of the technology used [17]. The production of NAbs results from the encounter between immune system B cells and viral glycoproteins, such as the spike protein [17]. This binding promotes the production of the antigen–BCR complex and signals to the major histocompatibility complex II (MHC II) on the surface of B cells, causing T-helper cells (CD4+) to recognize this binding and promote the release of IL-4, IL-5, IL-6, IL-21, and interferon-gamma (IFN-γ), which promote the differentiation of B cells into plasma cells responsible for the production of antibodies [18].

The elderly population naturally suffers from a decline in the maintenance of these vitamins and minerals. Aging is a natural factor that can lead to a decrease in the cutaneous synthesis of vitamin D3. However, exposure to sunlight is still an important factor for the production of this vitamin, as a previous study found that serum vitamin D3 concentration increased significantly in response to sun exposure in both younger and older individuals [19]. Factors other than low sun exposure and aging, such as poor diet and metabolic and absorption problems, can influence Vit D levels in the elderly [20]. In addition, studies in this population show that supplementation with these vitamins does not prevent immunosenescence associated with the antibody response, which involves the slow production and activation of cells and immunoglobulins in older and more debilitated individuals [21]. In view of this, the evaluation of the effect of vitamins and minerals on immunity is something that needs to be studied, especially with regard to the elderly population affected by the phenomenon of immunosenescence.

At the beginning of the pandemic, supplements were distributed to the general population in an inappropriate and arbitrary manner, with the aim of containing the spread of the virus. The most widely distributed supplements were Vit D and Zn. Misleading information during the process of prescribing and indicating these supplements and other drugs has the potential to jeopardize the well-being of the entire population, especially the elderly, who represent a vulnerable demographic group in this study. Currently, Vit D is no longer routinely prescribed; however, some studies highlight the benefits of these supplements in reducing mortality and disease severity from COVID-19, depending on the stage of the illness [22]. In this study, we assessed the neutralizing anti-SARS-CoV-2 antibodies of institutionalized elderly people in northeastern Brazil, and examined whether this immune response was related to Vit D and Zn levels.

## 2. Materials and Methods

**Figure 1:**
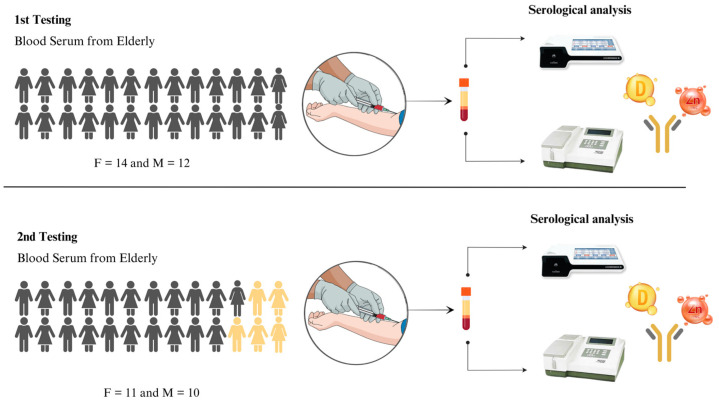
Stages in the methodology of the study presented. F = female and M = male. In the first test, we collected blood from the 26 participants and carried out laboratory analyses of neutralizing antibodies (NAbs), zinc (Zn), and vitamin D (Vit D), as well as administering the questionnaire. In the second collection, we carried out the same procedure, but with 21 participants who continued in the study.

The study was approved by the Research Ethics Committee (CAAE: 31018520.0.0000.5546) and focused on elderly people living in a retirement home located in the state of Sergipe, Brazil. A total of 26 people took part in the study, but five of them died during the study and were therefore excluded, as shown in Figure 1. The inclusion criteria consisted of living in a nursing home, not having a terminal illness, and formally agreeing to participate in the study. Initially, a questionnaire was administered to collect personal and clinical information about age, medications used, health conditions diagnosed, and the COVID-19 vaccination schedule.

### 2.1. Study Design

This study was conducted in two phases (Figure 1). The first phase occurred in August 2022, when the first serological sample was collected. Subsequently, the same participants selected in the first phase were targeted for a new collection, carried out after seven months, in March 2023, to observe whether there was a reduction in NAbs in this interval and to see whether levels of Vit D and Zn could interfere with the maintenance of antibodies. We considered a time difference of seven months between the dosages because this is the average time for the decay of antibodies in general [23]. In both phases, information was obtained made regarding the participants’ vaccination schedule and medication use.

### 2.2. Sample Collection and Laboratory Analysis

In order to analyze NAbs, Vit D, and Zn levels, it was necessary to collect biological material. This was performed by collecting venous blood from each patient, with a total of approximately 5 mL being deposited in a gel separator tube. This sample was then subjected to subsequent analysis using a semi-quantitative fluorescence immunoassay technique. NAbs and the Vit D were measured using the Ichroma™ II Reader (Boditech Med. Inc., Chuncheon-si, Gangwon-do, Republic of Korea) (https://www.boditech.co.kr/en/product/instruments/id/4, accessed on 10 January 2024) [24].

For NAbs, the ichroma COVID-19 nAb device was used (https://grupokovalent.com.br/reagente/ichroma-covid-19-nab/, accessed on 10 January 2024). The device can quantify the incidence of the signal, thus identifying the interaction between the antibodies present in the sample and their binding to the SARS-CoV-2 spike protein. A COI value of 30% or more is indicative of a reactive result, while a value < 30% is indicative of a non-reactive result. The detection of NAbs is based on the sandwich immunodetection technique, in which the SARS-CoV-2 antibodies present in the sample bind to the fluorescently labeled SARS-CoV-2 spike receptor-binding domain (RBD) antigen present in the detection buffer to form a complex in the sample, which migrates in the nitrocellulose matrix. The concentration of antibodies present in the sample is inversely proportional to the fluorescence signal, with higher antibody concentrations resulting in lower antigen detection and a correspondingly lower fluorescence signal. The manufacturer indicates a sensitivity of 95.8% and a specificity of 97% for this test.

To quantify Vit D, we used ichroma Vit D test (Boditech Med Inc., Gangwon-do, Republic of Korea) (accessed on 10 January 2024). This test is based on sandwich immunodetection, which allows the concentration to be detected by fluorescence. The buffer contained the antibodies that were responsible for detecting the potential antigens present in the sample. The combination of these proteins constituted the product deposited in the cassette, which contained a nitrocellulose matrix. The fluorescence signal was proportional to the number of antigen–antibody complexes formed. The reference values for serum Vit D levels were considered deficient (less than 10 ng/mL), insufficient (10–30 ng/mL), or sufficient (30–100 ng/mL), according to the manufacturer. The ichroma Vit D assay results were evaluated for precision and accuracy, demonstrating a recovery of 96% to 103% at varying concentrations of 25(OH)D2/D3. The overall precision was confirmed with three different batches, thereby ensuring consistency in the results. The data substantiate the suitability of the method for the precise determination of Vit D levels in human serum and plasma.

Zn analysis was conducted using a BioSystems BTS-310 spectrophotometer (https://www.medwrench.com/equipment/6103/biosystems-bts-310v, accessed on 10 January 2024), which employs an absorbance reader to indicate the presence of the sample in conjunction with the working reagent. The serum Zn level was measured using a spectrophotometric technique, whereby the mineral formed a red chelating complex with 2-(5-bromo-2-pyridylazo)-5-(N-propyl-N-sulfo-propylamino)-phenol. The increased absorbance of this complex was proportional to the concentration of Zn in the sample. The reading was obtained at a wavelength of 560 nm, with a blank reagent serving as the reference point. The reference values established by the reagent manufacturer for adults range from 46 to 150 µg/dL. 

### 2.3. Statistical Analysis and Data Visualization

Following the laboratory analysis of the samples, the results were tabulated using Microsoft Excel^®^ software (Microsoft 365 for Windows). The data were subsequently analyzed using IBM SPSS Statistics software (version 29.0, Windows). The initial evaluation of the sample distribution of the results obtained was conducted using a Welch’s *t*-test and paired-samples *t*-test to assess the results for NAbs, Vit D, and Zn between the two testing periods. A Circos^®^ graph (http://circos.ca/ (accessed on 29 January 2024)) was employed to illustrate the mean levels of Zn, Vit D, and NAbs, as well as the mean age and sex of the population. A *p*-value of 0.05 or less was considered statistically significant. All values following the mean with the symbol ± refer to the standard deviation (SD). The standard error was not used in this study. Due to the sampling methodology employed, the effect size (Cohen’s d) was utilized to assess the statistical power.

## 3. Results

### 3.1. Population Data Analysis

Ten women and eleven men participated in the study. The average age of the men was 70 ± 6 years, while that of the women was 75 ± 11 years. The participants had their vaccination schedule up to the fourth dose in the period of the first test, with the last update of the vaccines in March 2022, resulting in an interval of five months between vaccination and the first collection. In the second test, the vaccination schedule continued in December 2022, thus providing an interval of three months between vaccine update and the second test. Supplementation with Vit D and Zn was not part of the pharmacological scheme for the elderly individuals in question. 

Table 1 shows the quantitative results of each test conducted with the participants. It can be seen that, with regard to NAbs, there was a drop in the number of individuals with positive results in the second test compared to the first test. As for Vit D, none of the participants had very low levels, but some had insufficient Vit D, especially in the first test. As for Zn, a few individuals tested negative.

### 3.2. Association between the Serum Levels and the Sex of the Participants

An independent-sample Welch’s *t*-test was performed to investigate possible differences between the collected samples and sex. The results showed no statistically significant differences in NAbs in the first test (T1) between men (85 ± 29) and women (89 ± 19) (t(17.51) = 0.396, *p* > 0.05) (Cohen’s d = 0.18; very small effect), nor in the second test (T2) for men (82 ± 31) and women (57 ± 44) (t(15.91) = −1.5023, *p* > 0.05) (Cohen’s d = 0.70; moderate effect). Regarding Vit D levels, there was no statistically significant difference between men (31 ± 16) and women (39 ± 16) in the first test (T1) (t(18.95) = 1.056, *p* > 0.05) (Cohen’s d = 0.48; medium effect), nor in the second test (T2) between men (42 ± 11) and women (42 ± 16) (t(15.31) = 0.075, *p* > 0.05) (Cohen’s d = 0.04; very small effect). There were no statistically significant differences in Zn levels between men (103 ± 39) and women (116 ± 57) in the first test (T1) (t(15.86) = 0.607, *p* > 0.05) (Cohen’s d = 0.28; small effect), nor in the second test (T2) between men (M1 = 90.26 ± 39.46) and women (77 ± 29) (t(18.22) = −0.871, *p* > 0.05) (Cohen’s d = 0.39; small effect).

Furthermore, a paired *t*-test was performed with data stratified by sex to compare differences between the two test periods within each group. The results showed a statistically significant difference in Vit D production in men (T1 = 31 ± 17 vs. T2 = 42 ± 11) (t(10) = −2.906, *p* < 0.05) (Cohen’s d = 0.78; medium effect). For the other values, there was no significant difference for NAbs in men (T1 = 86 ± 29 vs. T2 = 82 ± 31) (Cohen’s d = 0.09; very small effect), nor for Vit D in women (T1 = 39 ± 16 vs. T2 = 42 ± 16) (Cohen’s d = 0.23; small effect) or Zn in men (T1 = 103 ± 39 vs. T2 = 90 ± 39) (Cohen’s d = 0.34; small effect). However, the mean NAbs levels in women (T1 = 89 ± 19 vs. T2 = 57 ± 44) (Cohen’s d = 0.99; large effect) and serum Zn levels in women (T1 = 116 ± 57 vs. T2 = 77 ± 29) (Cohen’s d = 0.92; large effect) showed notable differences. The small sample size (*n* = 21) may have limited the statistical power to detect any significant differences. Table 2 shows the NAbs, Zn, and Vit D results for sex at the two testing times.

### 3.3. Correlation between Zn, Vit D, and NAbs Production

Spearman’s correlation was used to ascertain whether a correlation existed between Vit D and Zn levels and the maintenance of NAbs at both time points. The correlation between variables was not statistically significant (*p* > 0.05). Fisher’s r-to-z transformation test indicated no association in the first test (z = 1.184; *p* > 0.05). Similarly, in the second test, there was no association with effect size (z = 0.585; *p* > 0.05).

### 3.4. Association between the General Population and Test Levels

A paired *t*-test was performed to analyze anti-SARS-CoV-2 NAbs and serum Vit D and Zn levels in a single group of elderly subjects at two time points separated by seven months. The results showed a statistically significant difference in Vit D levels during this interval (T1 = 35.70 ± 16 vs. T2 = 42 ± 13) (t(20) = −2.414, *p* < 0.05). Furthermore, although the results were not statistically significant (*p* > 0.05), a trend was observed during these months indicating a decrease in the levels of NAbs (T1 = 87 ± 24 vs. T2 = 70 ± 39) (Cohen’s d = 0.52; medium effect) and Zn (T1 = 109 ± 48 vs. T2 = 84 ± 35) (Cohen’s d = 0.62; medium effect).

### 3.5. Association between the Number of Vaccine Doses Used and NAbs Production

Welch’s *t*-test was conducted to examine the relationship between the number of vaccine doses (limited to four or five doses) received by the elderly individuals and the levels of NAbs. The results demonstrated a statistically significant correlation between the number of vaccine doses administered and NAbs levels. Individuals aged 65 and above who received four doses (*n* = 9) exhibited an average NAbs level of 56 ± 45, while those who received five doses (*n* = 12) demonstrated a significantly higher average of 95 ± 38 (Cohen’s d = 1.01; large effect).

## 4. Discussion

In this study, we analyzed the maintenance of NAbs over a seven-month period to evaluate immunity and the possible synergy with serum levels of Vit D and Zn in order to assess their possible influence on the behavior of adaptive immunity in institutionalized elderly patients in northeastern Brazil. Among the findings, a decline in NAbs against SARS-CoV-2 was observed in the interval between tests, which corroborates studies in the literature that report the decrease in NAbs over time [25]. However, it is important to emphasize that the immune response mediated by memory cells plays a role, because even with the reduction in NAbs, the levels of these antibodies were still above the minimum expected value, as the study cohort had already received at least two doses of the COVID-19 vaccine, which would be conducive to the stimulation of memory immunity [4].

Microelements in the serum can be important in helping the immune system combat the worsening of diseases [26]. Studies have shown that supplementation with micronutrients such as Vit D and Zn contributes to a satisfactory prognosis in the elderly [27]. Our study involved non-hospitalized patients who generally had sufficient levels of Vit D and Zn. In line with the present study, Davoudi et al. (2021) also found no association between Vit D levels and a lower risk of adverse clinical outcomes for COVID-19 such as severity of infection or duration of hospitalization [27]. With these findings, it becomes evident that there is no direct correlation between the levels of NAbs and the maintenance of Vit D titration. An analysis of the existing literature indicates that there is a positive modulation of the immune system when a patient infected with SARS-CoV-2 has Vit D levels between 30 and 100 ng/mL [28].

A meta-analysis of 25 randomized clinical trials (RCTs) found that Vit D supplementation appears to be beneficial in reducing intensive care unit (ICU) admission due to COVID-19 and in reducing mortality in patients who were deficient in this vitamin [29]. A randomized study assessed the production of NAbs in individuals with previous SARS-CoV-2 infection. Participants received the following interventions over a 42-day study period: hydroxychloroquine (400 mg followed by 200 mg/day), povidone–iodine throat spray (three times a day, approximately 270 μg/day), oral ivermectin (12 mg, single dose), Zn + vitamin C (80 mg zinc sulfate, 500 mg vitamin C/day), and exclusively vitamin C (500 mg/day). The group receiving Zn + vitamin C showed a greater NAbs response compared to the other intervention groups [30].

This population is classified as being at risk of developing disease owing to immunosenescence, which is caused by immune aging. This aspect influences the production of humoral immunity and defense effector cells against pathogen invasion and stimulates the production of memory cells from vaccines. We suggest that immunosenescence contributes to the decline in NAbs observed in the study population over seven months, similar to the findings in the literature [31]. A study by Kim et al. (2022) concluded that in a hospitalized population, people over the age of 50 were more likely to have NAbs decreasing more quickly compared to the younger group [32]. In other words, although the severity of the disease caused an increase in NAbs levels, they fell rapidly. In addition, compared to our cohort, the study led by Neuhann et al. (2022) evaluated the immunogenicity of elderly people over 74 years of age when vaccinated with different vaccines and concluded that, despite the increase in NAbs caused by booster doses, the production of these antibodies may have been limited by immunosenescence in the target population [33].

A gender analysis revealed that women exhibited a more pronounced decline in NAbs titration at T1 and T2 than men. This condition is associated with the postmenopausal physiological process, which is characterized by a reduction in estrogen levels. Estrogen is classified as an ally of antiviral, hormonal, and inflammatory responses, acting as an immune protector and stimulating antibody production [34,35]. In a previous study by our research group, higher levels of acute-phase antibodies were observed in women aged >40 years. However, no significant differences were identified between sexes with regard to memory antibodies. It should be noted that this study was conducted before the implementation of the vaccination program, and memory antibodies were solely derived from a previous infection [36]. On the other hand, another study by the group, carried out with the academic population of the Federal University of Sergipe, found no significant difference between memory antibody and acute-phase antibody production by sex [37]. Notably, the methodology employed in the aforementioned studies was to investigate the presence and titration of acute-phase and memory antibodies.

A previous study by our group showed another association with antibody production, where our group identified an increase in the intense production of antibodies in a population in the same state with each vaccine intervention imposed by the Brazilian vaccination schedule [38]. The population’s vaccination schedule allows for flexible measures to contain the virus. This makes it possible to address social aspects that make a difference in the lives of the elderly, especially individuals who are institutionalized [39]. Maintaining immunity through vaccination helps to combat the emergence of new strains. In this way, it enables the population to return to all work activities, especially for the study participants, since isolation itself as a way of avoiding contamination by the virus can result in cognitive, nutritional, and even functional decline in the elderly [39]. 

A growing body of evidence suggests that Zn plays a role in immune function and may have beneficial effects during the ongoing pandemic. The results indicated a positive correlation between serum levels within the reference range and the prevention of disease progression [38]. However, we did not find any relationship between Zn and NAbs. Notably, the target group in this study included asymptomatic patients. With regard to Zn, a reduction in serum levels was observed in the second test, which may have been due to dietary changes and a reduction in daily intake. This finding is consistent with the data reported by Knez et al. (2023) [40]. Additionally, some drugs in the class of antihypertensives, anticonvulsants, and corticosteroids may have contributed to this reduction [41,42].

This study has some limitations, and one of them is the size of our sample. It is worth noting that a sample calculation was not possible due to the lack of information about the population size of nursing home residents in the city in question. In addition, this population is difficult to access, as some individuals are not in an adequate cognitive state or do not have family members present on a daily basis to provide consent for the study, and many also choose not to participate due to personal issues. In Brazil, there are not many studies in this population, reflecting the importance of our research. Another limitation is, the absence of reverse transcription polymerase chain reaction (RT-PCR) test and/or antigen test and clinical tests that make it possible to assess active infection, since patients could be infected without showing symptoms of COVID-19 disease, representing asymptomatic patients. The absence of antibody titers, such as acute-phase antibodies and memory antibodies for SARS-CoV-2, could be related to NAbs levels. The loss of five participants during the study was not related to COVID-19 but to age. In line with this, patients were unable to indicate whether they were infected during the collection period due to the lack of tests to verify the infection.

The study has some strengths, such as access to a more restricted population, which is the elderly population living in nursing homes. Age is an important factor for severe cases of COVID-19, which emphasizes the importance of monitoring the immune systems of this population. In addition, we evaluated the synergy of micronutrients with the response of these elderly people in a post-vaccination period, finding an absence of synergy. Finally, we monitored microelements and NAbs against SARS-CoV-2, which are important parameters for maintaining and caring for the health of these elderly people, enabling health interventions when necessary.

## 5. Conclusions

The results point to a decline in NAbs, even with satisfactory levels of Vit D and Zn, in the population studied. Based on our results, we suggest that there is no synergism between microelements and the maintenance of NAbs in the elderly population. A greater reduction in NAbs was observed in female participants compared to male participants. In this study, we draw attention to new studies aimed at evaluating the effects of vitamin and mineral supplementation on the robustness of the immune system in elderly individuals. In this way, we seek to overcome the limitations we encountered when carrying out this study, especially in relation to the sample size and the immunological parameters applied. Finally, we emphasize that monitoring this population and evaluating their vitamin and mineral levels is necessary, especially when exposed to infectious and aggressive diseases such as COVID-19.

## Figures and Tables

**Table 1:** Evaluation of patients COI. COI: Cut Off Index.

NAbs			Vit D			Zn	
Reference	Positive	Negative	Deficient	Insufficient	Sufficient	Positive	Negative
	≥30%	<30%	<10 ng/mL	10–30 ng/mL	30–100 ng/mL	46–150 µg/dL	<46 µg/dL
T1	20 (95%)	1 (4.8%)	0 (0%)	11 (52%)	10 (48%)	19 (90%)	2 (9.5%)
T2	16 (76%)	5 (24%)	0 (0%)	4 (19%)	17 (81%)	18 (86%)	3 (14%)

**Table 2:** The relationship between females (F) and males (M) in neutralizing antibodies (NAbs), vitamin D (Vit D), and zinc (Zn). The average of the levels between two time points was evaluated, where the first test is T1 and the second test is T2.

	NAbs (%)		Vit D (ng/mL)		Zn (µg/dL)	
	M	F	M	F	M	F
T1	86 ± 29	89 ± 19	31 ± 17	39 ± 16	103 ± 39	116 ± 57
T2	82 ± 31	57 ± 44	42 ± 11	42 ± 16	90 ± 39	77 ± 29

## Data Availability

The data will be provided by contacting the corresponding authors.
